# Rapid Single Particle Atmospheric Solids Analysis Probe-Mass Spectrometry for Multimodal Analysis of Microplastics

**DOI:** 10.1021/acs.analchem.2c04345

**Published:** 2022-12-22

**Authors:** Clementina Vitali, Hans-Gerd Janssen, Francesco Simone Ruggeri, Michel W. F. Nielen

**Affiliations:** † Wageningen Food Safety Research, Wageningen University & Research, Akkermaalsbos 2, 6708 WB Wageningen, The Netherlands; ‡ Laboratory of Organic Chemistry, Wageningen University, Stippeneng 4, 6708 WE Wageningen, The Netherlands; § Unilever Foods Innovation Centre − Hive, Bronland 14, 6708 WH Wageningen, The Netherlands; ∥ Physical Chemistry and Soft Matter, Wageningen University, Stippeneng 4, 6708 WE Wageningen, The Netherlands

## Abstract

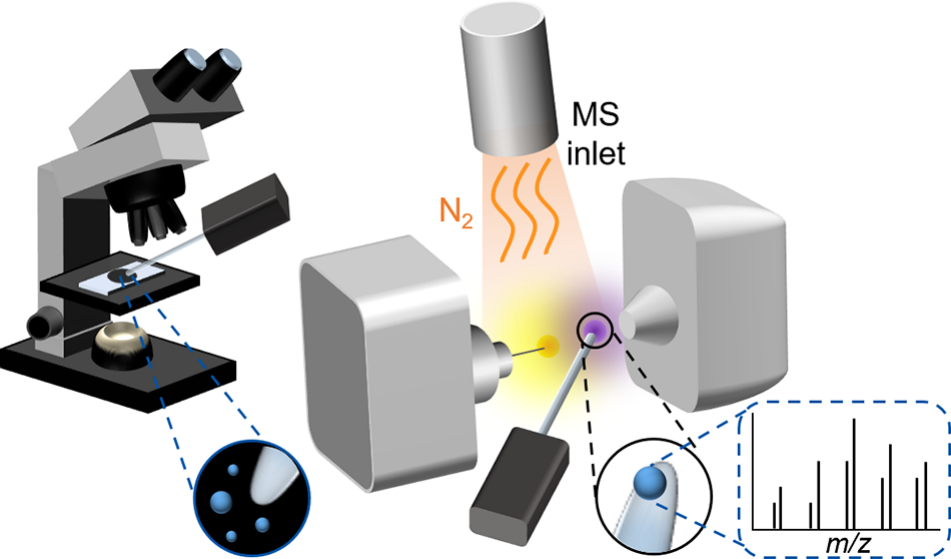

Despite mass spectrometry (MS) being proven powerful for the characterization of synthetic polymers, its potential for the analysis of single particle microplastics (MPs) is yet to be fully disclosed. To date, MPs are regarded as ubiquitous contaminants, but the limited availability of techniques that enable full characterizations of MPs results in a lack of systematic data regarding their occurrence. In this study, an atmospheric solid analysis probe (ASAP) coupled to a compact quadrupole MS is proposed for the chemical analysis of single particle microplastics, while maintaining full compatibility with complementary staining and image analysis approaches. A two-stage ASAP probe temperature program was optimized for the removal of additives and surface contaminants followed by the actual polymer characterization. The method showed specific mass spectra for a wide range of single particle MPs, including polyolefins, polyaromatics, polyacrylates, (bio)polyesters, polyamides, polycarbonates, and polyacrylonitriles. The single particle size detection limits for polystyrene MPs were found to be 30 and 5 μm in full scan and selected ion recording mode, respectively. Moreover, results are presented of a multimodal microplastic analysis approach in which filtered particles are first characterized by staining and fluorescence microscopy, followed by simple probe picking of individual particles for subsequent analysis by ASAP-MS. The method provides a full characterization of MP contamination, including particle number, particle size, particle shape, and chemical identity. The applicability of the developed multimodal method was successfully demonstrated by the analysis of MPs in bioplastic bottled water.

## Introduction

Microplastics (MPs) are defined as plastic products — primary MPs — or debris — secondary MPs — with size between 1 and 5000 μm.^1^ In recent years, MPs have gathered the growing attention of the scientific community^2^ and the general public as proof of their presence has been found in the most remote environmental compartments,^3,4^ food,^5^ and even human tissues,^6^ thus being recognized as ubiquitous contaminants. Yet, available analytical methods for the analysis of MPs are laborious and time intensive,^7^ resulting in a lack of data regarding MP occurrence.^5^ The tedious duration and complexity of protocols for the analysis of MPs are mainly due to the multidimensionality of the data necessary for a full physicochemical MP characterization. Number of particles, particle size and size distribution, particle shape and shape distribution, color, chemical identity, and particle mass are the parameters commonly addressed in MP analysis. Particle size and shape are of particular relevance as preliminary studies suggest they have an effect on the absorption, translocation, and ultimately the toxicity of MPs.^8−12^ Therefore, analysis aimed at producing data eligible for environmental or food safety risk assessment must be able to characterize MPs at the single particle scale.

To date, Fourier transform infrared spectroscopy (FTIR) and Raman spectroscopy are the most promising and widespread analytical techniques for MP identification.^7^ The combination of optical microscopy and vibrational spectroscopy (μFTIR and μRaman) provides most of the relevant information about MP contamination, including particle number, particle size distribution, particle shape, chemical composition, and identification. However, single particle spectroscopic methods are challenged by producing concentration data on a weight basis. Moreover, the chemical identification can be compromised by the overlap in the spectra of dyes and pigments added during manufacturing.^13^

Mass spectrometry (MS) in combination with pyrolysis and gas chromatography (Py-GC/MS) has been adopted as an alternative approach for the analysis of MPs.^14−16^ The technique is capable of chemical characterization of synthetic polymers independently from the sizes of the plastic fragments contaminating the sample and provides a mass-based concentration of plastic material. An alternative to Py-GC/MS, ambient MS, allows the analysis of liquid and solid samples without any sample treatment or chromatographic separation.^17−19^ Direct analysis in real time (DART) MS has been tested for the analysis of MPs, showing promising results in the quantification^20^ and fingerprinting^21^ of polymeric materials. However, in both Py-GC/MS and ambient MS, as samples are typically analyzed in bulk, most of the information relevant for the full physicochemical characterization of MPs gets lost in the microfurnace or probe upon pyrolysis.

Atmospheric solids analysis probe (ASAP)^22^ MS showed promising results in the past for the analysis of synthetic polymers^23−28^ but, to our knowledge, has never been evaluated for the analysis of MPs. ASAP-MS enables the analysis of solid and liquid samples that are deposited on a probe and inserted directly into the ionization chamber. In there, a heated nitrogen flow promotes the desolvation and/or thermal degradation of the sample, and a corona discharge initiates the ionization via the generation of radical species and/or via water cluster-mediated proton transfer, yielding radical cations and/or protonated molecules of the sample.

In this study, a transportable MS system featuring an ASAP source and a single quadrupole mass analyzer^29^ is evaluated for the analysis of MPs. The technique allows MS-based rapid single particle analysis of MPs. The probe temperature program was optimized for overcoming matrix interference and allows the identification of synthetic polymers belonging to different chemical families. Finally, a multimodal approach was investigated in which the newly developed technique was combined with a selective staining technique and fluorescence microscopy, resulting in a comprehensive characterization of MPs, yielding particle number, size, shape, and MS-based chemical characterization.

## Experimental Section

### Chemicals and materials

Polystyrene (PS) analytical standards with certified particle sizes (5, 10, 30, 100, 150, 200 μm) and microparticles based on polymethacrylate (PMMA) with certified particle sizes (60, 100 μm) were purchased from Sigma-Aldrich (Schnelldorf, Germany). Polyacrylonitrile (PAN) 50 μm, polyamide-6 (PA 6) 55 μm, poly(ethylene terephthalate) (PET) 300 μm, and poly(hydroxy butyrate)/poly(hydroxy valerate) 2% biopolymer (PHB) 300 μm were sourced from Goodfellows (Hamburg, Germany). Polyamide-46 (PA 46), poly(butylene terephthalate) (PBT), polycarbonate (PC), and polypropylene (PP) were purchased from Goodfellows (Hamburg, Germany) as rods and ground in-house to obtain microsized particles. Ultrahigh molecular weight polyethylene (PE) 40–48 μm was purchased from Sigma-Aldrich. Blue PE microspheres 125–150 μm were supplied by Cospheric (Santa Barbara, California, United States). PS pellets were sourced from a local plastic production plant and ground in house. Tween20 and Nile Red were purchased from Sigma-Aldrich. Nile Red stock solution was prepared in acetone (Actu-All Chemicals, Oss, The Netherlands) and diluted in ethanol (Supelco). Raspberry and pomegranate flavored vitamin drink and bottled water were purchased from a local store. Sealed ends soda glass capillaries were sourced by Fisher Scientific (Loughborough, UK).

### Instrumentation

Single MP particles were sampled with sealed soda glass capillaries under an Olympus (Hamburg, Germany) BX51 microscope, equipped with Olympus 4×, 10×, 20×, and 40× objectives, an Olympus U-RFL-T UV lamp, and a band-pass filter characterized by excitation and emission wavelengths of respectively 460–490 nm and >515 nm. Pristine MPs were sampled in brightfield mode, while Nile Red stained MPs were sampled in fluorescence mode. An Olympus SC50 camera installed on the microscope was used for image acquisition. The images, acquired by cellSens software (Olympus), were saved in tagged image file format (.tif).

Single particle MP analysis was performed on a RADIAN ASAP instrument (Waters Corporation, Manchester, UK) consisting of a single quadrupole mass analyzer equipped with a horizontal loading fixed geometry ASAP source. The ASAP-MS settings were optimized as follows: nitrogen gas flow was set at 3.0 L min^–1^; gas temperature was programmed as two isothermal heating steps of 375 and 600 °C, which were kept constant for 1 and 2 min, respectively. The corona current was +3 μA, and MS data were acquired at a cone voltage of 15 V. Data were acquired in full scan positive ion mode (*m*/*z* 50–1200) and occasionally by selected ion recording (SIR). Mass Lynx v4.2 software (Waters Corporation) was used for instrument control and MS data analysis.

### Methods

Prior to ASAP analysis, each probe capillary was exposed to a cleaning step (600 °C nitrogen gas during 60 s) in order to degrade and remove any impurities on the probe surface and to provide the elevated temperature of the glass capillary for adhesion of the MP during single particle picking under the microscope. For the development of the single particle method, MPs were dispersed on a glass slide and singularly sampled with the glass capillary under the microscope at 10× magnification. The capillary bearing a single MP particle was then immediately inserted in the ASAP ion source. Glass capillaries were disposed and not reused.

**Figure 1:**
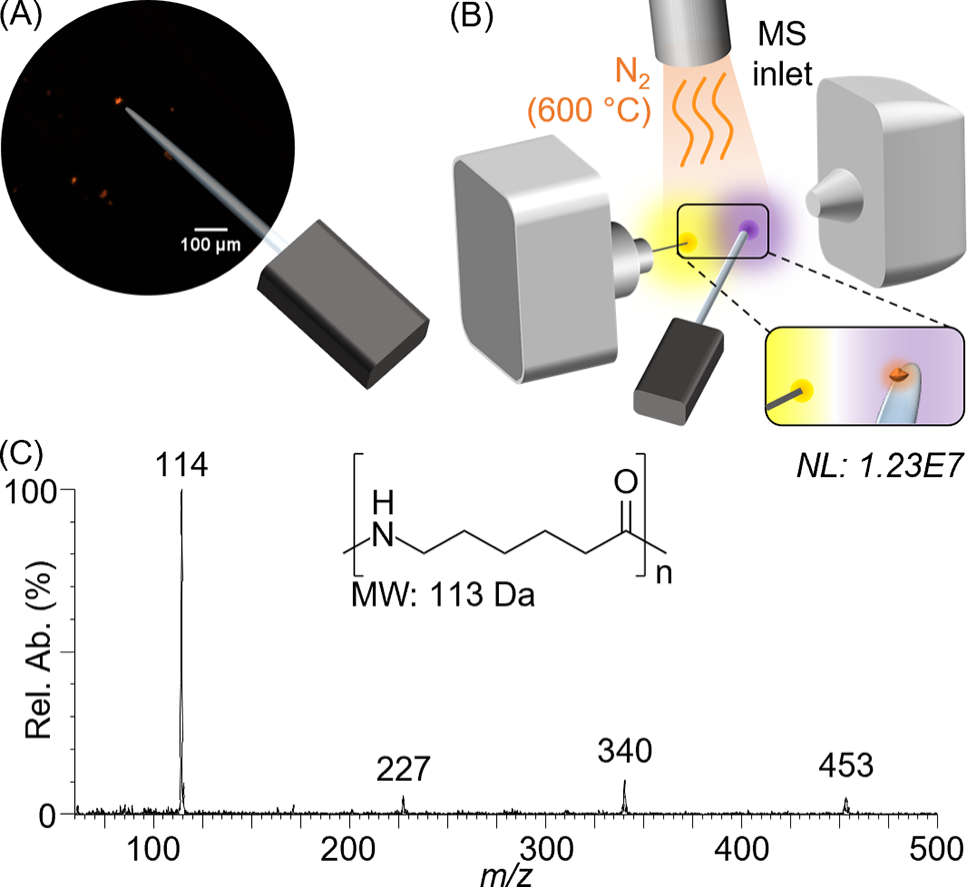
Workflow for multimodal analysis of microplastics (MPs) using ASAP-MS. (A) Nile Red stained MPs are collected on a filter and image analyzed by a fluorescence microscope. A single MP is sampled with the glass probe. (B) The probe is inserted into the ASAP ion source where a first thermal cleanup stage is performed, and then, by rising the nitrogen flow temperature, thermal degradation is promoted. The resulting fragments are ionized by corona discharge under ambient conditions. (C) ASAP mass spectra of a stained single polyamide 6 particle sampled from the filter (A).

To demonstrate the multimodal compatibility of the ASAP-MS method with selective staining and fluorescence microscopy of MPs, particles were dispersed in ultrapure water containing 0.2% Tween20, stained with Nile Red,^30^ and analyzed by ASAP-MS according to the schematic workflow shown in Figure 1.

The applicability of the said multimodal characterization method was tested by the analysis of the content of a 500 mL bottle of mineral water. Particle size distribution was obtained based on the measurement of the major Feret diameter; the items were classified based on their shapes as previously reported.^31^

### Performance Characteristics of the Developed ASAP-MS Method

Procedural blanks were run to assess the absence of memory effects between the ASAP-MS analyses. Furthermore, to assess the repeatability of the ASAP polymer spectra, hence the suitability of the technique for fingerprint based identification, the analysis of each polymer was performed in triplicate: for each repetition, a new particle was sampled on a clean glass capillary. PS analytical standards with certified particle sizes were analyzed to assess the method detection limit as per particle size. Mixed MPs from different polymers were sampled on the same capillary and analyzed simultaneously to assess the performance of the ASAP-MS instrument in the simultaneous characterization of multiple particles and to investigate the formation of any in-source artifacts. The performance characteristics of the Nile Red staining and fluorescence microscopy-based quantification protocol have been fully assessed and described in a dedicated manuscript.^30^

## Results and Discussion

### ASAP-MS Method Optimization

**Figure 2:**
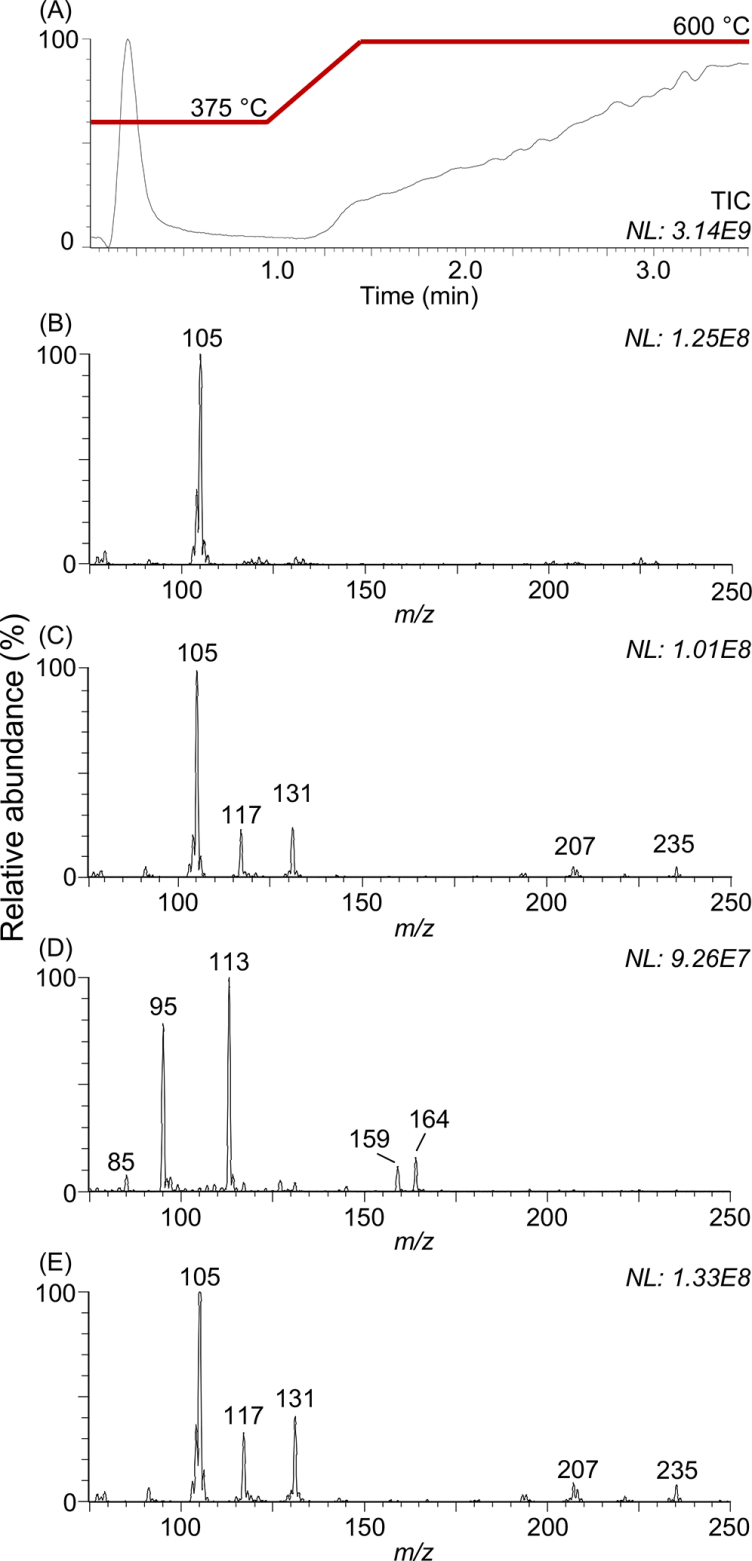
(A) Chronogram and temperature profile of atmospheric solids analysis probe-mass spectrometry analysis of polystyrene (PS) single particle MP. (B) Background subtracted mass spectrum of a sample of a pristine PS MP during the cleanup stage at 375 °C. (C) Background subtracted mass spectrum of the same sample but at the polymer characterization stage at 600 °C. (D) Background subtracted mass spectrum of the PS MP covered by a food matrix (a commercial multivitamin drink) at the cleanup stage 375 °C. (E) Background subtracted spectra of the same PS in a food matrix (sample acquired at the 600 °C stage).

The optimization of the instrumental conditions was performed using PS microspheres having diameters of 150 μm. The nitrogen gas temperature program consisted of two isothermal heating steps and a 3 min 30 s analysis time (Figure 2A). It was developed with the aim of performing first a cleanup for the removal of free styrene monomers from the polymer MP (*m*/*z* 105 in Figure 2B) and any residual MP surface contamination from the sample matrix, followed by a second thermal degradation step (Figure 2C) optimized for polymer characterization. For the optimization of the gas temperature during the cleanup stage, tests were carried out at 150, 300, 350, 375, and 400 °C, but a setting of 375 °C was showed to be the optimum condition. The second isothermal heating stage was set at the maximum temperature allowed by the instrument, i.e., 600 °C, in order to promote the pyrolysis of the synthetic polymers. The great efficacy of the rapid cleanup step was confirmed by the analysis of PS MPs in a complex food matrix: 2 μL of a commercial multivitamin beverage were pipetted on a probe capillary bearing a 150 μm PS single particle. Figure 2D and E shows the resulting mass spectra. Interferents from the degradation of the sample matrix are clearly observed in the spectrum acquired during the cleaning step, while the mass spectra acquired at the 600 °C stage, in contrast, are free of interferences. PS is simply identified by its protonated ion at *m*/*z* 105 and comparison with the spectrum of pristine PS (Figure 2C). Since the ASAP-MS temperature-programmed analysis allows the detection of polymer additives present in and on samples of synthetic polymers,^23,32^ this strategy opens new possibilities for future studies of, e.g., MPs isolated from environmental and food samples, also from a toxicological point of view.

### Method Performance

The spectra obtained by running procedural blanks were analyzed to exclude any memory effect happening between consecutive analyses.

The repeatability of the ASAP-MS spectra was fit-for-purpose regarding the presence of characteristic ions, while the ion ratios showed some variability, particularly in the analyses of PA 4,6 and PC single particles.

To assess the method detection limit as per particle size, analytical standards of PS single particle MPs with certified diameter sizes of 150, 100, 30, 10, and 5 μm were analyzed by ASAP-MS following probe picking from a glass slide under a microscope. Among the tested materials, 30 μm PS beads showed to be the smallest single particles detectable in the TIC mass spectra acquired in full scan mode with a peak to peak signal-to-noise ratio (S/N) of 4.5. For the detection of even smaller MPs, a 3 min isothermal (600 °C) SIR method including the three characteristic ions of PS at *m*/*z* 105, 117, and 131 (Figure 2C) was tested. Single 10 μm PS beads were detectable in SIR mode with a peak to peak S/N of 250. Single 5 μm PS beads analyzed in SIR returned a peak to peak S/N of 2.7.

### MPs Analysis

To assess the extent of the applicability of ASAP-MS for the analysis of a wide range of MPs, the method was tested on a set of ubiquitous nonpolar polymers used in packaging (PE, PP, and PS), on different condensation polymer families (polyamides, polyesters, polycarbonates) and on additional polymers of common use such as PMMA used in screens and acrylic painting, PAN used as a synthetic textile fiber, and PHB used in, e.g., biobased and biodegradable plastic items.^33^ Each of the analyzed plastic polymers, 12 in total, resulted in characteristic mass spectra, suitable for fingerprint analysis.

Previously, ASAP-MS was shown to be able to ionize both polymers having a low proton affinity, such as PE^27^ and PP,^25,32^ and other nonpolar polymers,^26^ e.g., PS.^28^ The same was applied to single particle MP analyses of PE, PP, and PS in this study, without the addition of any salt to promote ionization (Figures S1–S3). The ASAP mass spectra of different types of PE (Figure S2) are characterized by distributions of ions having a repeating mass difference of 28 Da, in accordance with the molecular weight of the ethylene monomer. Consistent with previous work,^25,32,34^ the analysis of PP MPs resulted in multiple ion distributions each having a mass difference of 42 Da (Figure S3), the molecular weight of propylene monomer. The highest abundance in the ASAP mass spectra of single particle PS is observed for the protonated monomer at *m*/*z* 105, while *m*/*z* 104 shows the radical cation of the monomer. The abundant ions at *m*/*z* 117 and 131 were shown to be characteristic as well, while *m*/*z* 207, 235, and 312 are present at a lower intensity only (Figure S1A). The ASAP-MS spectra we obtained by the analysis of PS differ from previous literature,^28^ by the low intensity of higher molecular weight oligomers. The method was tested on a second PS sample consisting of a ground PS pellet, resulting in reproducible spectra showing the characteristic peaks at *m*/*z* 105, 117, 131, 207, 235, and 312 (Figure S1B). The higher relative abundance of low molecular weight degradation products versus PS oligomers in the spectra can mostly be ascribed to the low proton affinity of bigger apolar oligomers and a consequent higher likeliness to ionize the first ones rather than the latter. Moreover, it should be noted that a relatively high residence time in the heated chamber of the ASAP source can promote the further degradation of the pyrolysis products into smaller molecules.

A specific feature of MS versus alternative techniques is its capability to differentiate straightforward between different MPs belonging to the same polymer family. For example, different single particle MPs belonging to the polyamides class, such as PA 6 and PA 4,6 were analyzed by single particle ASAP-MS. PA 6 showed a reproducible ion series having a mass difference of 113 Da, starting from *m*/*z* 114, that could be attributed to the protonated monomer and oligomers (Figure S4). In contrast, the ASAP mass spectra of PA 4,6 are characterized by ions from the protonated repeating unit(s) at *m*/*z* 199 and 397, and abundant fragments thereof at *m*/*z* 115 and 313 (Figure S5).

Similarly, in the polyester family, single particle PET, PBT, and PHB were analyzed by ASAP-MS, and a polycarbonate (PC) was investigated as well. The ASAP mass spectra of PET showed two distributions of ions having a mass difference of 192 Da, in accordance with the molecular weight of the repeating ethylene terephthalate unit (Figure S6). The ion series starting at *m*/*z* 193 represents protonated intact oligomers, while the series starting at *m*/*z* 149 represents the loss of ethylene oxide thereof. Single particle PBT could be nicely distinguished from other polyesters as demonstrated by the two ion series having a mass difference of 220 Da, which could be attributed to the repeating unit of PBT (Figure S7). The ion series starting at *m*/*z* 221 represents protonated intact oligomers, while the series starting at *m*/*z* 149 represents the loss of butylene oxide thereof. The abundant ion at *m*/*z* 423 may represent a protonated cyclic dimer. PHB showed ion series with a difference of 86 Da, which could be attributed to the molecular weight of hydroxybutyrate (Figure S8).

In contrast, the ASAP mass spectra of PC showed two high intensity ions at *m*/*z* 509 and 763, that could be attributed to the protonated dimer and trimer of bisphenol A (Figure S9).

PMMA and PAN were also detectable by ASAP-MS analysis. Single particle PMMA MP analysis showed the protonated monomer at *m*/*z* 101 and an abundant fragment at 73 (Figure S10). PAN showed multiple ion series featuring the characteristic mass difference of 53 Da, which could be attributed to the molecular weight of acrylonitrile (Figure S11).

**Figure 3:**
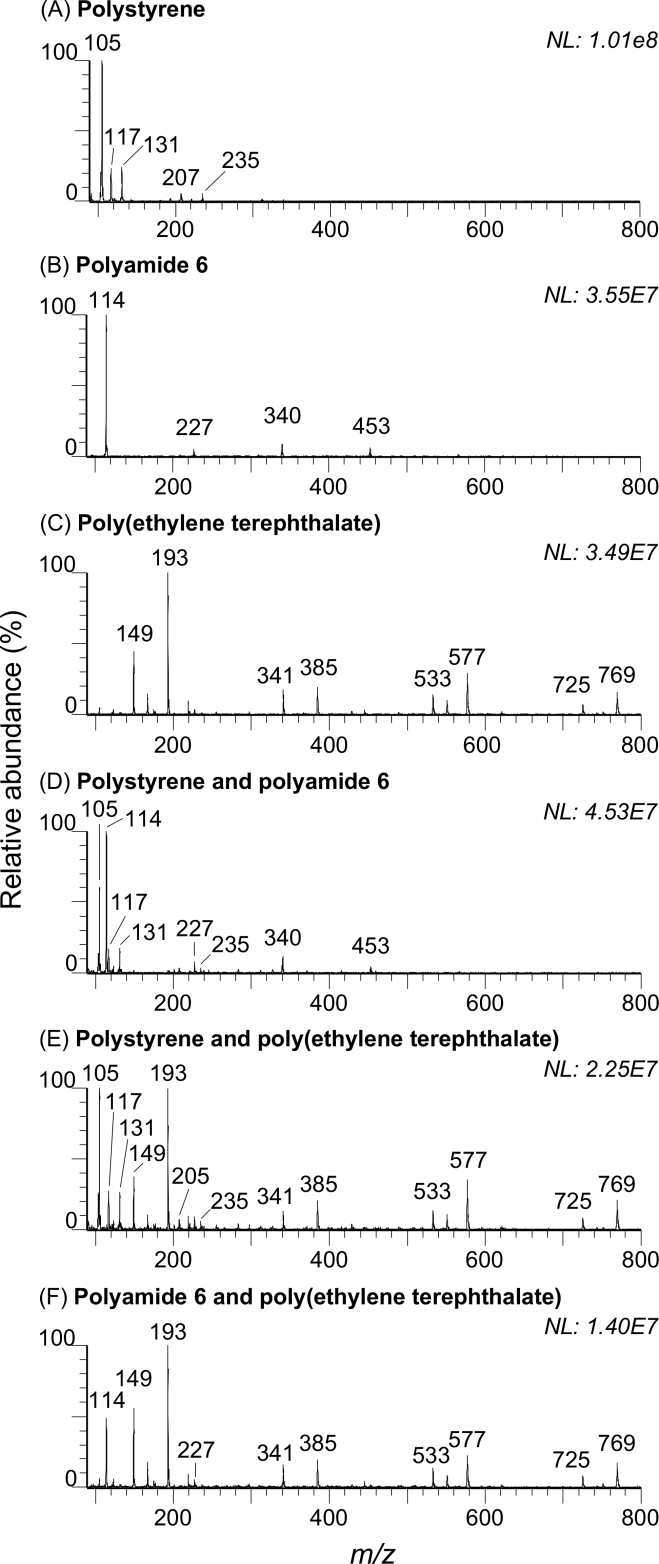
Atmospheric solids analysis probe background subtracted mass spectra of single polystyrene (A), polyamide 6 (B), and poly(ethylene terephthalate) (C). MPs and combinations thereof in pairs (D, E, F). The comparison between single polymer spectra and mixed polymer spectra allows the evaluation of ASAP-MS for the simultaneous characterization of different polymers and suggests that the observed oligomer ions are the products of thermal degradation rather than in-source artifacts caused by clustering.

Figure 3 shows a comparison of the spectra of PS, PET, and PA and their combinations in pairs, obtained by loading single probes with two MPs of different chemical composition. When two polymers are analyzed (Figure 3D–F), ions from the combined individual spectra are only observed, without any additional degree of complexity, allowing the easy identification of both polymers at once. This nicely demonstrates the potential for intended mixture analyses of multiple particles in order to increase sample throughput in chemical analysis of MPs. Moreover, this result is interesting for the assessment of the occurrence of in-source ion clustering events. Most of the analyzed single particle MP, in fact, showed ion distributions simply differing by the monomer repeating unit, which could represent both polymer fragments produced during thermal degradation and in-source artifacts caused by clustering of, for example, residual monomers in the MP polymer standards. Even though the latter is unlikely to occur at the ionization conditions of an ASAP source, the use of a portable instrument with a relatively small footprint for the transition of atmospheric pressure to vacuum led us to verify this hypothesis. The absence of additional ions in the spectra obtained by the analyses of multiple polymers clearly suggests that no ion clustering occurs among the products of thermal degradation and/or in-source fragmentation. In that case, additional artifact ions should have been observed in the spectra, caused by aggregation of fragments from the degradation of the two different plastic MPs.

### Multimodal Characterization of MPs

The single particle ASAP-MS analysis of MPs was then combined with Nile Red staining and fluorescence microscopy, thus building a truly multimodal characterization method able to yield particle number, size, shape, and MS-based chemical characterization of MPs. To assess the compatibility of Nile Red staining and fluorescence microscopy-based detection and quantification with ASAP-MS analysis, MPs from all the investigated polymers were suspended in water having 0.2% Tween20, incubated with Nile Red dye, then isolated and analyzed by ASAP-MS. No interference was observed from the dye nor from the additive (Figures S12–20), underlining the full compatibility, except for PE and PP: in the spectra of the nonpolar PE and PP (which are hard to ionize by any MS method), an ion series with a mass difference of 44 Da replaced the expected signal (Figures S21 and S22). This is clearly caused by (fragmentation of) the Tween20 additive, that contains ethylene oxide oligomers in its chemical structure, and produces a ASPA-MS spectrum characterized by multiple ion distributions with a difference of 44 Da (Figure S23). However, the addition of a surfactant to the MP suspension is crucial for a quantitative approach to the analysis of MPs, as it promotes the homogeneous dispersion of the particles and inhibits their interaction with the walls of the glassware, preventing their loss along the analysis procedure.^30^ Nevertheless, several surfactants are commercially available and can be tested for their efficacy in promoting satisfactory recovery values while minimizing interference with polymer identification.

### Applicability of Multimodal Analysis of MPs in a Real Sample

**Figure 4:**
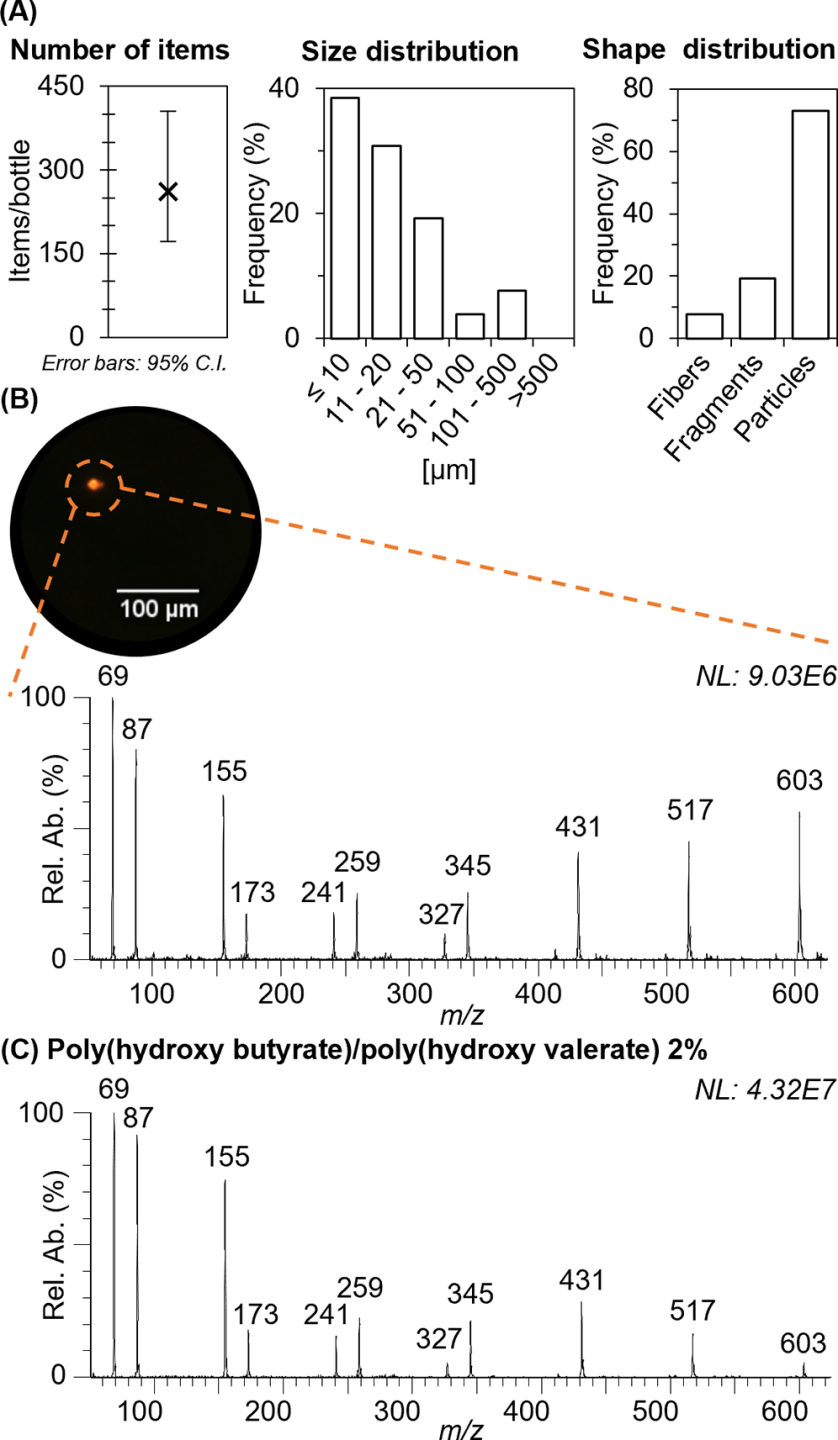
Multimodal characterization of MPs in drinking water, results. (A) Quantitative and qualitative analysis based on Nile Red staining and fluorescence microscopy of MPs filtered from bottled water purchased at a local store. (B) Fluorescence picture of one of the isolated and stained particles and its ASAP-MS background subtracted spectrum, allowing its chemical identification. (C) ASAP-MS background subtracted spectrum of standard poly(hydroxy butyrate).

Finally, the method was tested on MPs isolated from bottled water purchased in a local store. The entire content of the bottle (500 mL) was incubated with Nile Red and filtered; then, the isolated particles were analyzed by fluorescence microscopy. Quantitative and qualitative results, including the number of stained items, their size distribution, and shape distribution, are reported in Figure 4A. Figure 4B shows one of the stained MPs detected by fluorescence microscopy. Following picking by the ASAP probe under the microscope and subsequent MS analysis, the mass spectrum shown in Figure 4B was obtained. The spectrum clearly shows a characteristic ion series featuring a repeating unit mass of 86 Da that nicely compares with the single particle ASAP-MS spectrum of our standard PHB MPs (Figure 4C). This multimodal characterization approach enables all the relevant information to describe MP contamination. Combining, for the first time, the capabilities of fluorescence imaging for the quantitative and qualitative analysis of particles and the capabilities of MS for single particle chemical identification. Chemical identification at the single particle scale is necessary for an accurate physicochemical characterization of MP contamination, as it allows one to only include confirmed synthetic polymer data and exclude irrelevant particle data from the MP number and size distributions obtained from the microscopy image analysis.

## Conclusions

The capability and potential of ASAP-MS for rapid single particle chemical analysis of a wide range of MPs have been demonstrated. The characteristic mass spectra obtained enable a rapid identification of the synthetic polymer(s) in the MPs in particles as small as 30 or 5 um, depending on the acquisition mode. A two-stage probe temperature program was optimized for the ASAP-MS analysis of MPs in a matrix and was found to effectively cope with a complex sample matrix such as a vitamin drink. The developed ASAP-MS method is compatible with current staining procedures and fluorescence microscopy for most of the MPs tested, thus paving the way for a full multimodal characterization of MPs, including the number of particles, their size and shape distributions, and MS-based chemical characterizations, relevant for environmental and food safety risk assessment.

## Supporting Information Available

Main characteristic ions in single particle ASAP-MS of microplastics; ASAP-MS spectra of single microplastic particles (polystyrene; polyethylene; polypropylene; polyamide 6; polyamide 4,6; polyethylene terephthalate; polybutylene terephthalate; polyhydroxy butyrate; polycarbonate; poly(methyl methacrylate); polyacrylonitrile); ASAP-MS spectra of Nile Red stained single microplastic particles (polystyrene; polyamide 6; polyamide 4,6; polyethylene terephthalate; polybutylene terephthalate; polyhydroxy butyrate; polycarbonate; poly(methyl methacrylate); polyacrylonitrile; polyethylene; polypropylene); and ASAP-MS spectra of Tween 20 0.2% in ultrapure water (PDF)
