# Supplementary material for: Rapid Single Particle Atmospheric Solids Analysis Probe-Mass Spectrometry for Multimodal Analysis of Microplastics
Source: Anal Chem. 2022 Dec 22;95(2):1395–401. doi: 10.1021/acs.analchem.2c04345 (PMC9850409; doi:10.1021/acs.analchem.2c04345)

Supporting information

Rapid Single Particle Atmospheric Solids Analysis Probe – Mass Spectrometry for Multimodal Analysis of Microplastics

Clementina Vitali[1,2]*, Hans-Gerd Janssen[2,3], Francesco Simone Ruggeri[2,4]*, Michel W. F. Nielen[1,2].

[1] Wageningen Food Safety Research, Wageningen University & Research, Akkermaalsbos 2, 6708 WB Wageningen, The Netherlands.

[2] Wageningen University, Laboratory of Organic Chemistry, Stippeneng 4, 6708WE, Wageningen, The Netherlands.

[3] Unilever Foods Innovation Centre – Hive, Bronland 14, 6708 WH Wageningen, The Netherlands.

[4] Wageningen University, Physical Chemistry and Soft Matter, Stippeneng 4, 6708WE, Wageningen, The Netherlands.

Table of Contents

Supplementary tables 3

Table S1 Main characteristic ions in single particle ASAP-MS of microplastics 3

Supplementary figures 4

Figure S1 Polystyrene 4

Figure S2 Polyethylene 4

Figure S3 Polypropylene 5

Figure S4 Polyamide 6 5

Figure S5 Polyamide 4,6 5

Figure S6 Poly(ethylene terephthalate) 6

Figure S7 Poly(butylene terephthalate) 6

Figure S8 Poly(hydroxy butyrate) 6

Figure S9 Polycarbonate 7

Figure S10 Poly(methyl methacrylate) 7

Figure S11 Polyacrylonitrile 7

Figure S12 Nile Red stained polystyrene 8

Figure S13 Nile Red stained polyamide 6 8

Figure S14 Nile Red stained polyamide 4,6 8

Figure S15 Nile Red stained poly(ethylene terephthalate) 9

Figure S16 Nile Red stained poly(butylene terephthalate) 9

Figure S17 Nile Red stained poly(hydroxy butyrate) 9

Figure S18 Nile Red stained polycarbonate 10

Figure S19 Nile Red stained poly(methyl methacrylate) **10**

Figure S20 Nile Red stained polyacrylonitrile **10**

Figure S21 Nile Red stained polyethylene **11**

Figure S22 Nile Red stained polypropylene...**11**

Figure S23 Tween20 0.2% in ultrapure water (1 μL)...**11**

Supplementary tables

Table S1 Main characteristic ions in single particle ASAP-MS of microplastics

Polymer	Characteristic ions (*m/z*)
Polystyrene	105; 117; 131; 207; 235; 312
Polyethylene	Multiple distributions, Δ = 28 Da
Polypropylene	Multiple distributions, Δ = 42 Da
Polyamide 6	114; 227; 340; 453
Polyamide 4,6	115; 199; 287; 313; 353; 397
Poly(ethylene terephthalate)	149; 193; 341; 385; 533; 577; 725; 769
Poly(butylene terephthalate)	149; 167; 221; 275; 369; 423; 441; 495; 607; 661; 715; 881; 935
Poly(hydroxy butyrate)	69; 87; 155; 173; 241; 259; 327; 345; 341; 517; 603
Polycarbonate	509; 527; 763
Poly(methyl methacrylate)	59; 69; 73; 85; 101
Polyacrylonitrile	107; 148; 160; 174; 201; 213; 215; 227; 254; 268; 280; 307; 321; 335; 274

Supplementary figures

ASAP-MS spectra of single microplastic particles sampled from a glass slide under a microscope.

Figure S1 Polystyrene. (A) Standard polystyrene microspheres. (B) Polystyrene particle obtained by in-house grinding of polystyrene pellet.

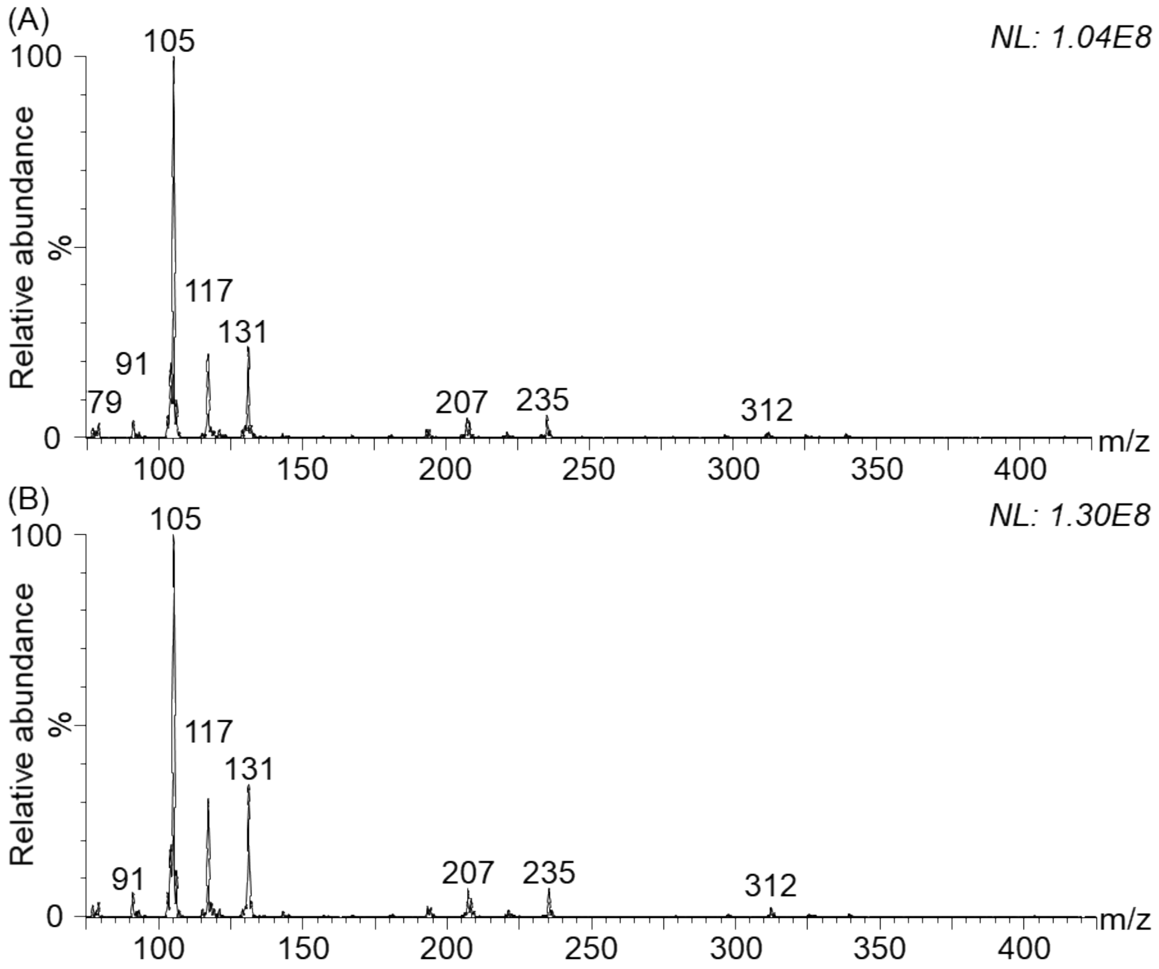

Figure S2 Polyethylene

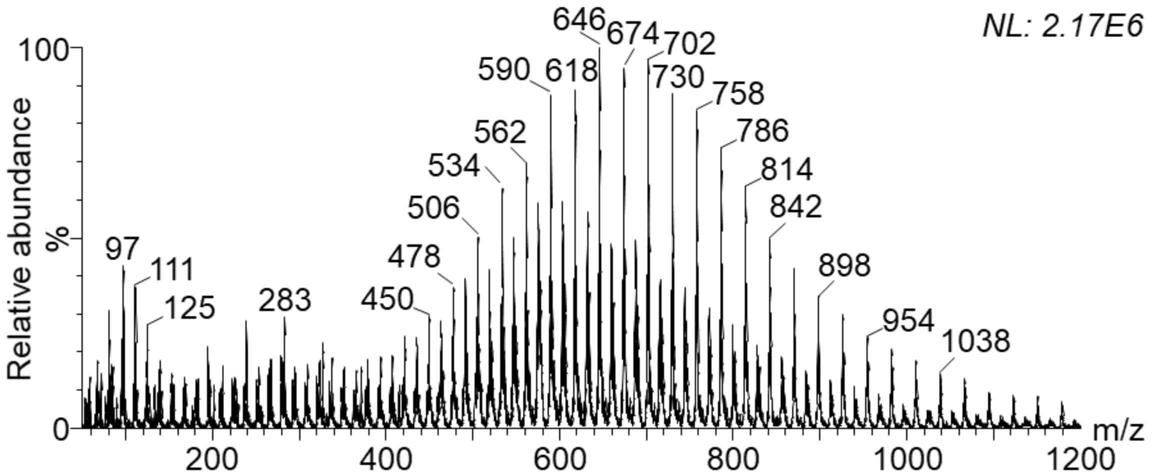

Figure S3 Polypropylene

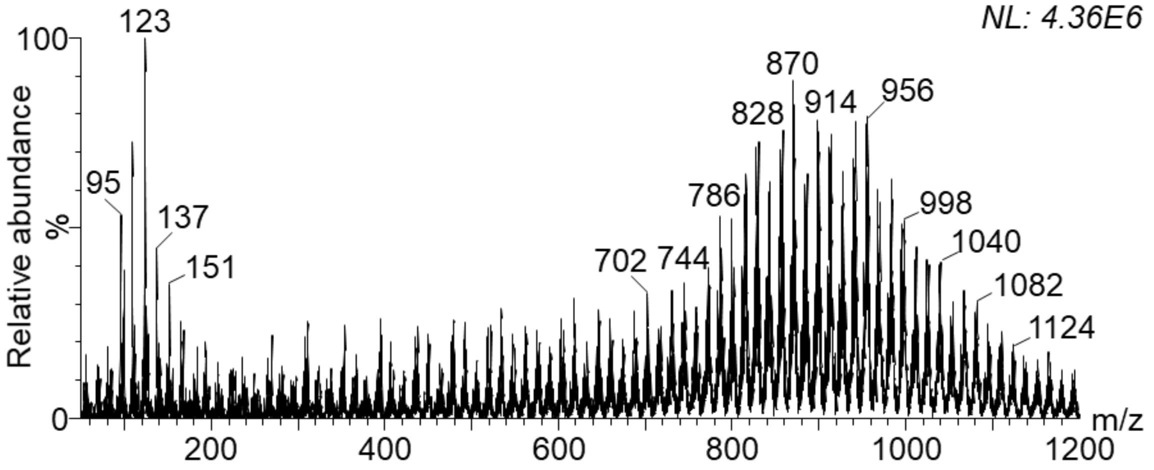

Figure S4 Polyamide 6

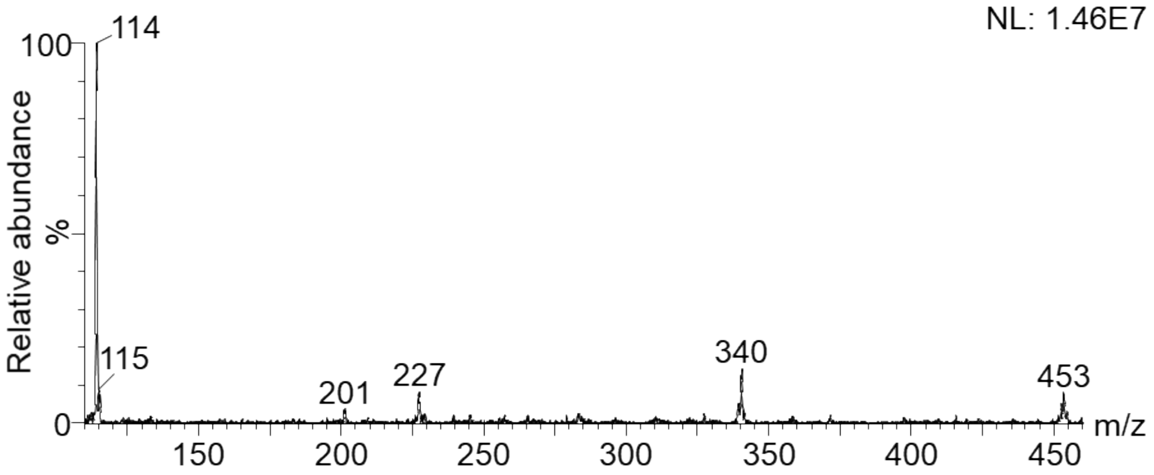

Figure S5 Polyamide 4,6

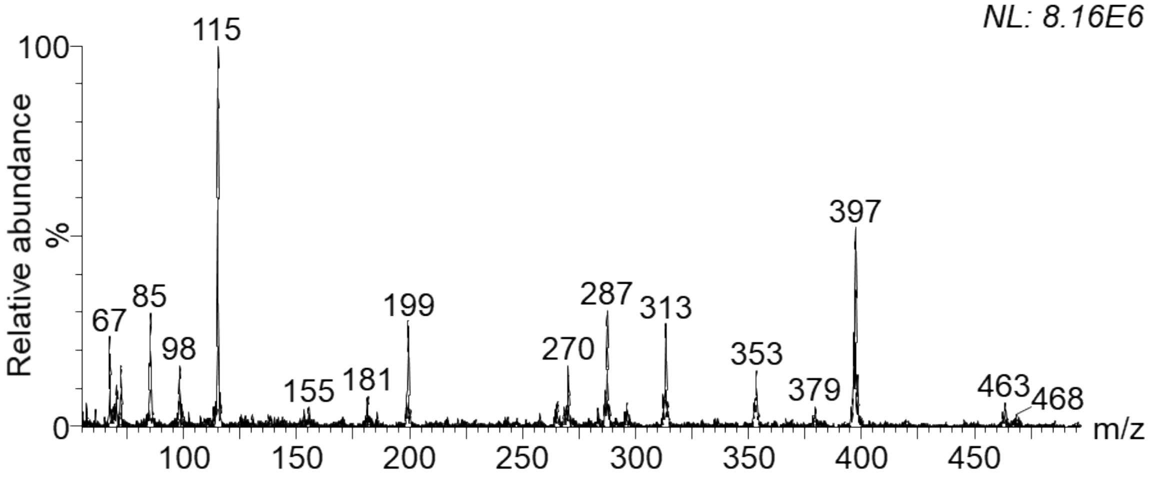

Figure S6 Poly(ethylene terephthalate)

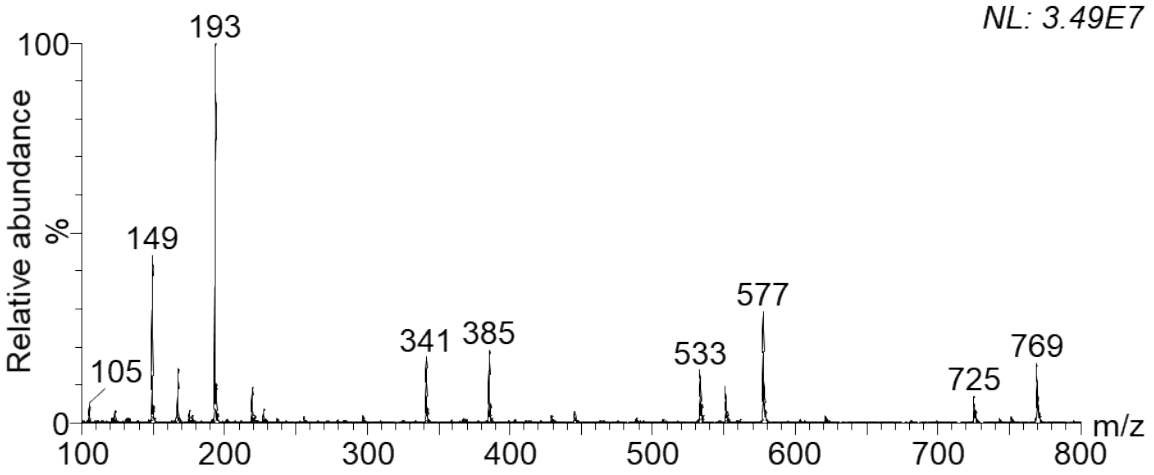

Figure S7 Poly(butylene terephthalate)

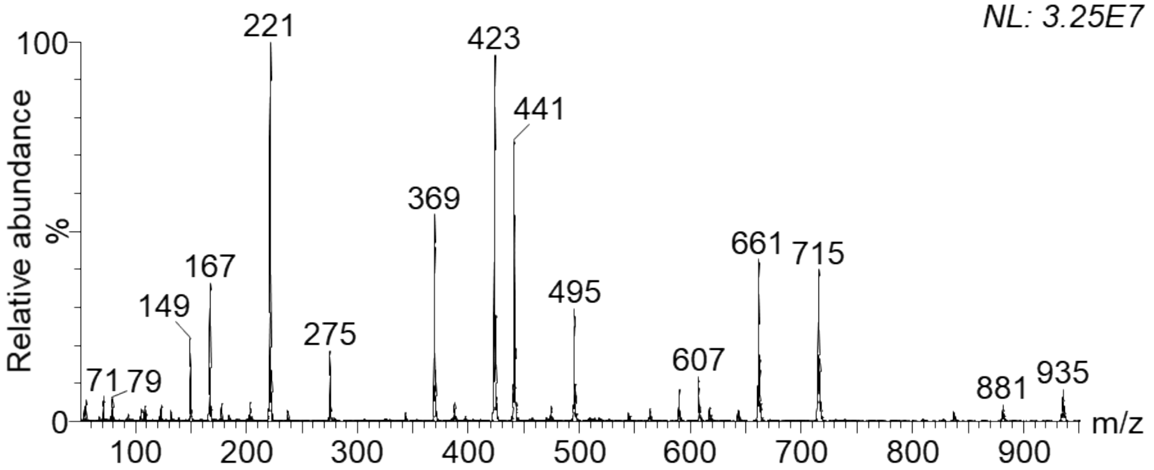

Figure S8 Poly(hydroxy butyrate)/poly(hydroxy valerate) 2%

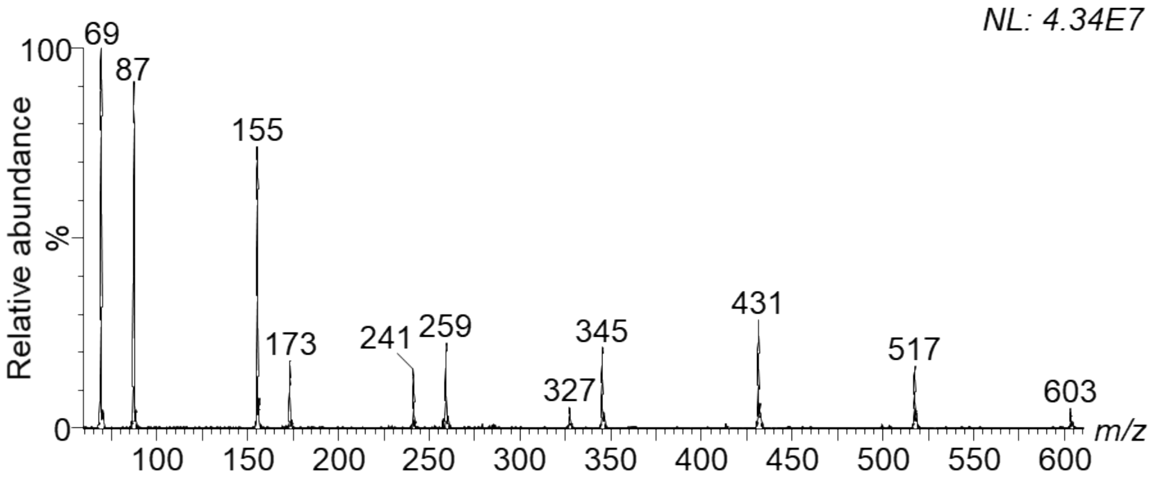

Figure S9 Polycarbonate

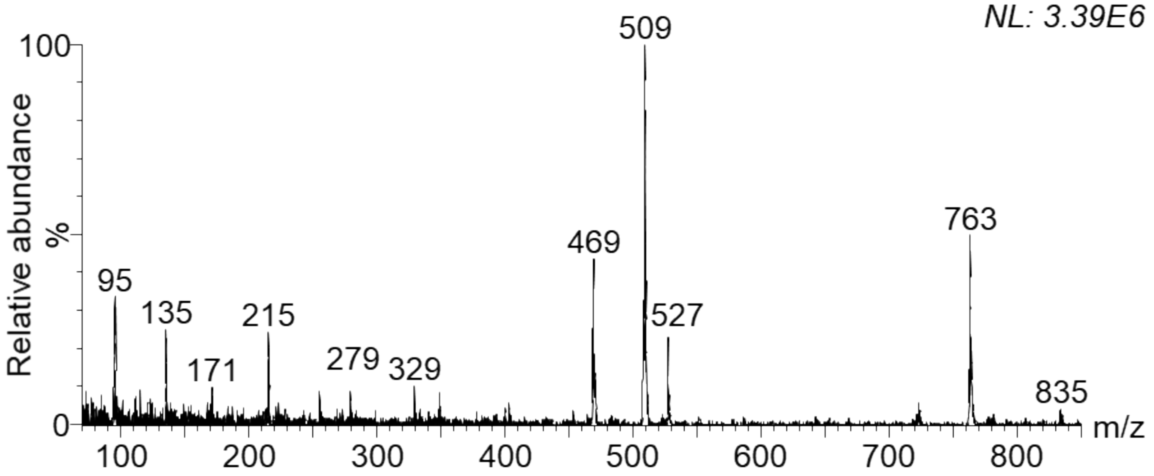

Figure S10 Poly(methyl methacrylate)

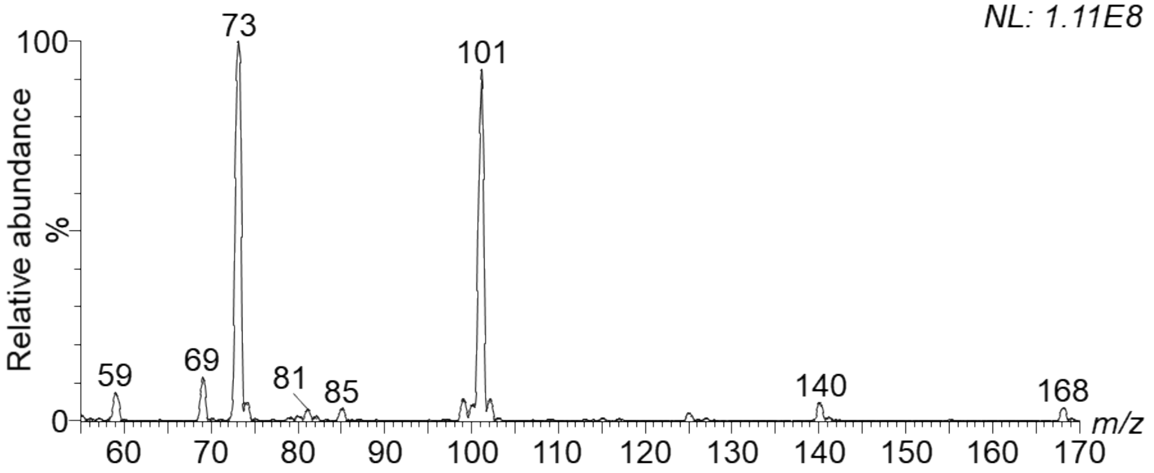

Figure S11 Polyacrylonitrile

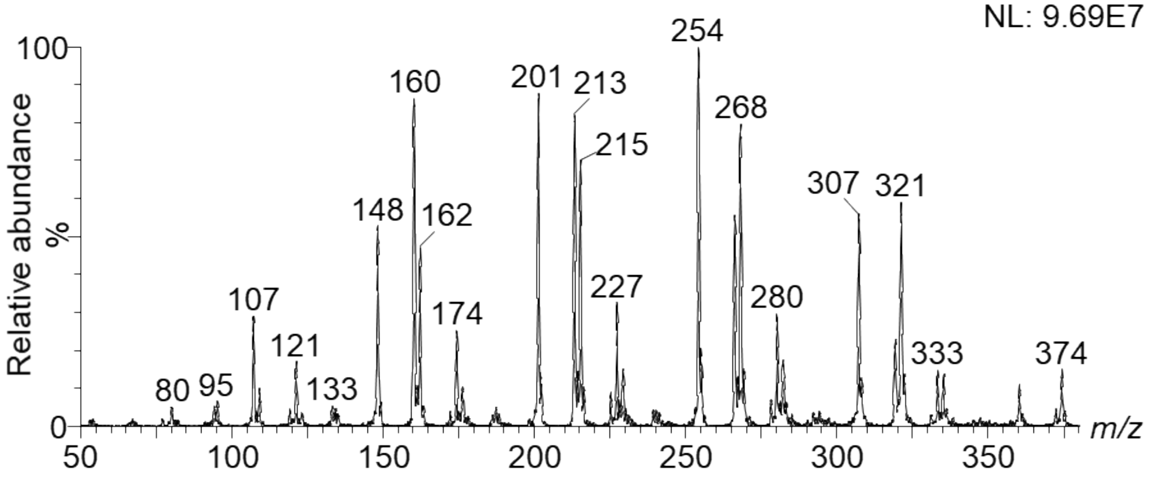

ASAP-MS spectra of single microplastic particles sampled from a glass slide under a fluorescence microscope. Prior analysis, the particles were dispersed in ultrapure water and Tween20 and stained with Nile Red.

Figure S12 Nile Red stained polystyrene

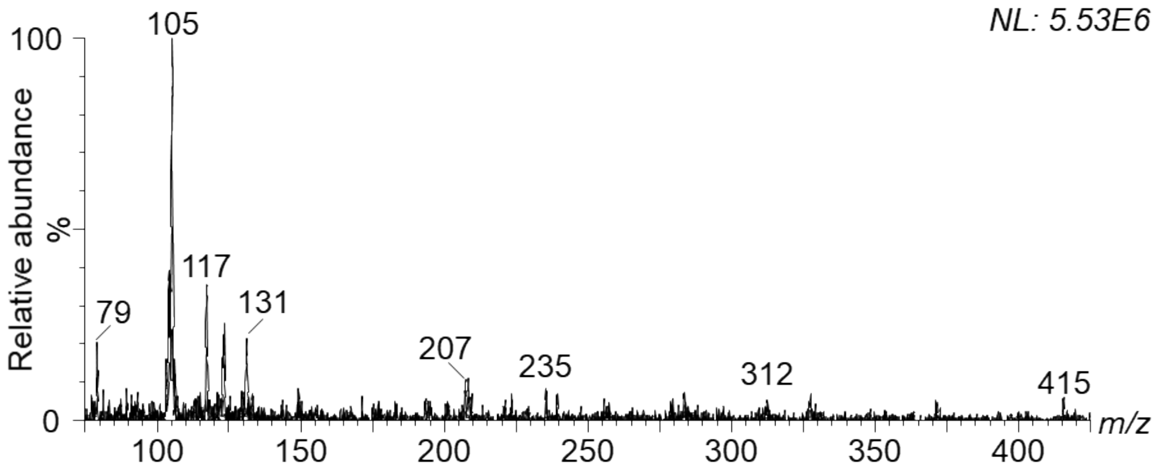

Figure S13 Nile Red stained polyamide 6

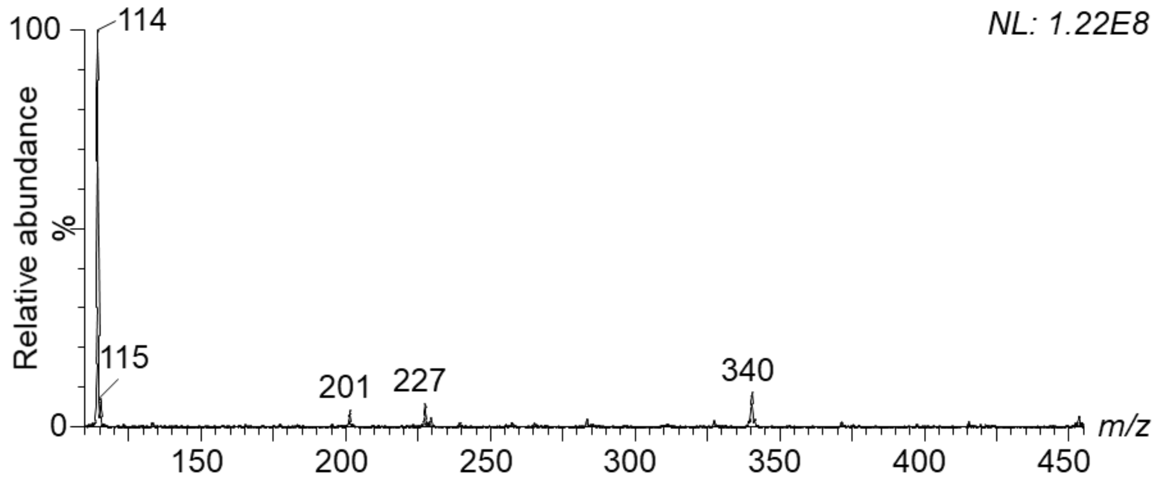

Figure S14 Nile Red stained polyamide 4,6

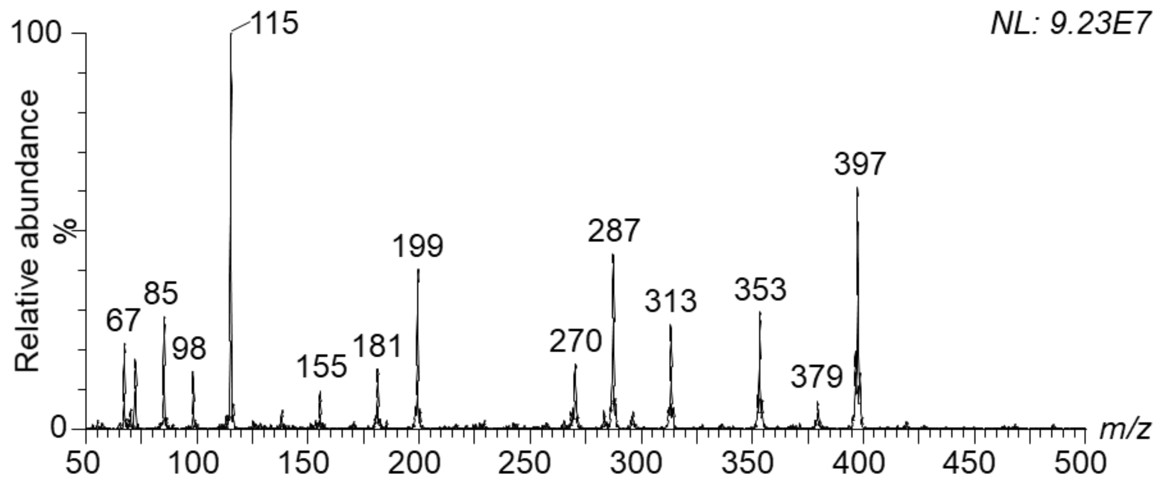

Figure S15 Nile Red stained poly(ethylene terephthalate)

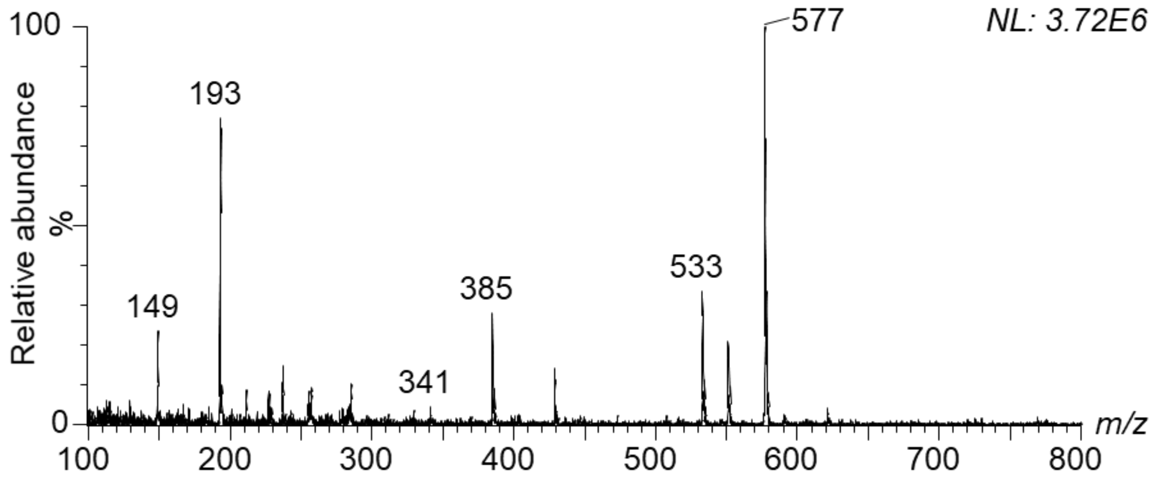

Figure S16 Nile Red stained poly(butylene terephthalate)

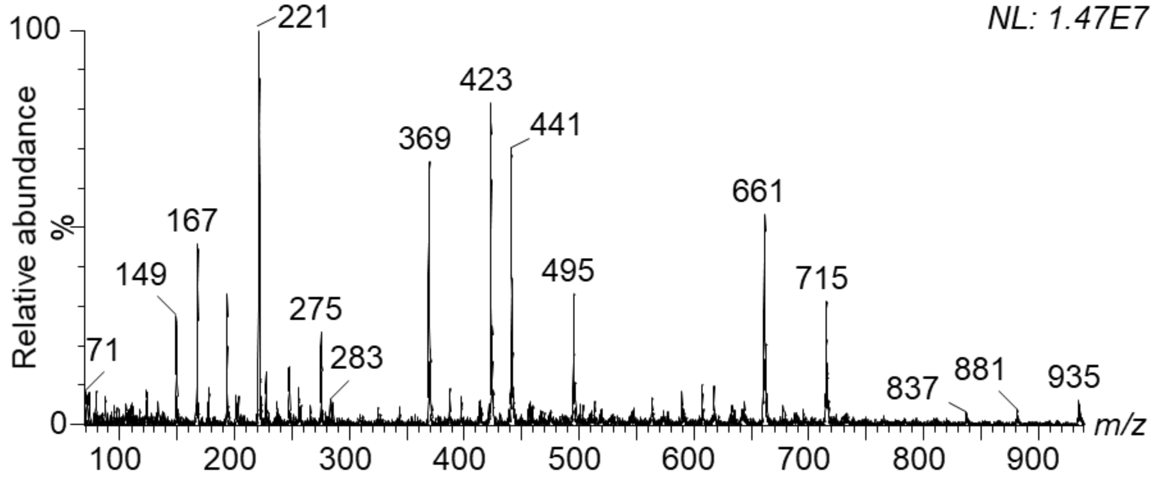

Figure S17 Nile Red stained poly(hydroxy butyrate)

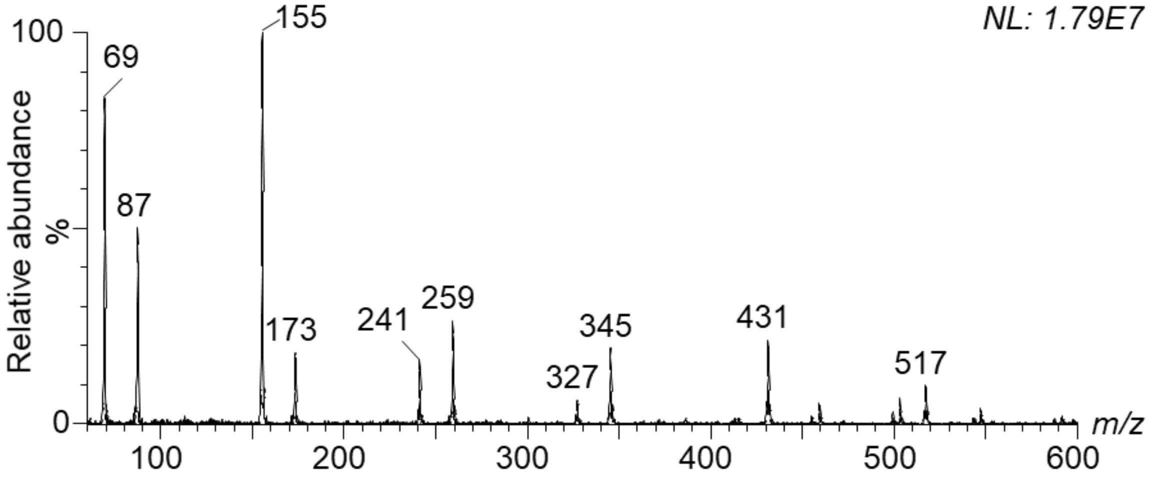

Figure S18 Nile Red stained polycarbonate

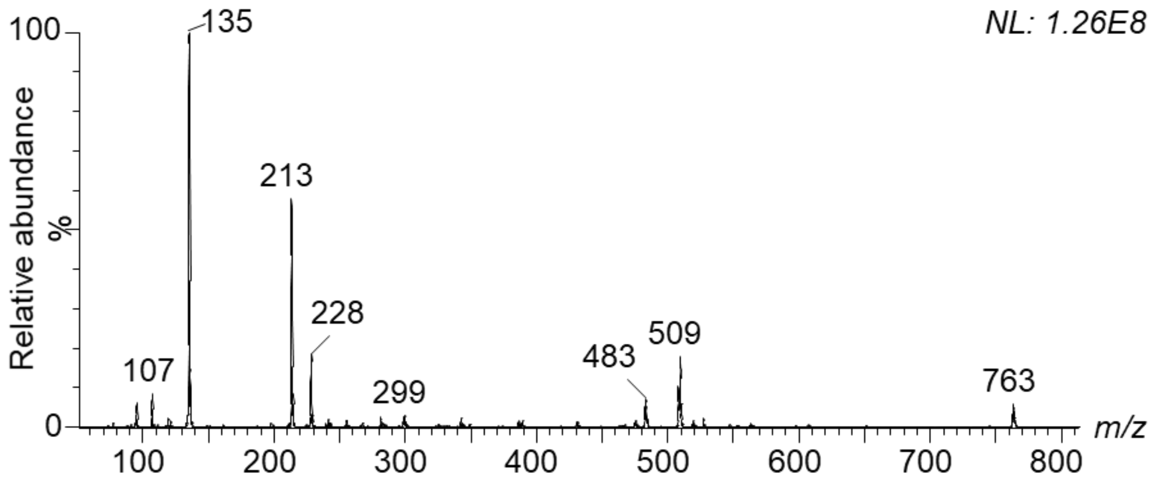

Figure S19 Nile Red stained poly(methyl methacrylate)

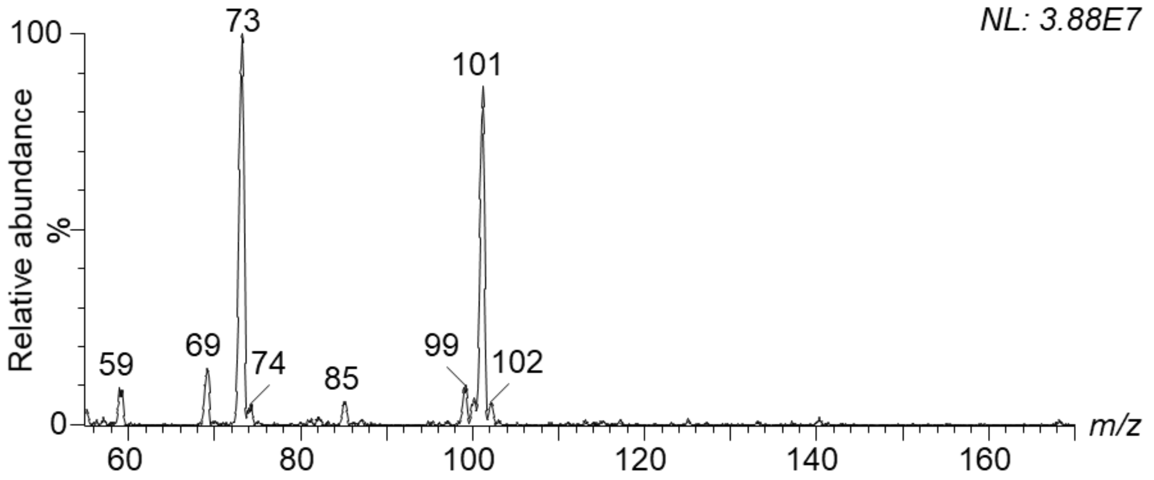

Figure S20 Nile Red stained polyacrylonitrile

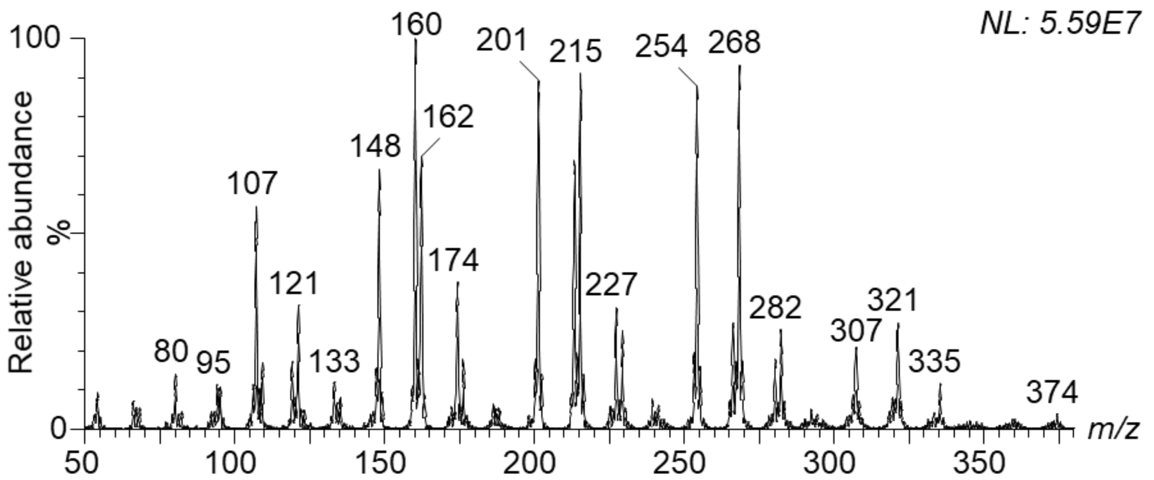

Figure S21 Nile Red stained polyethylene

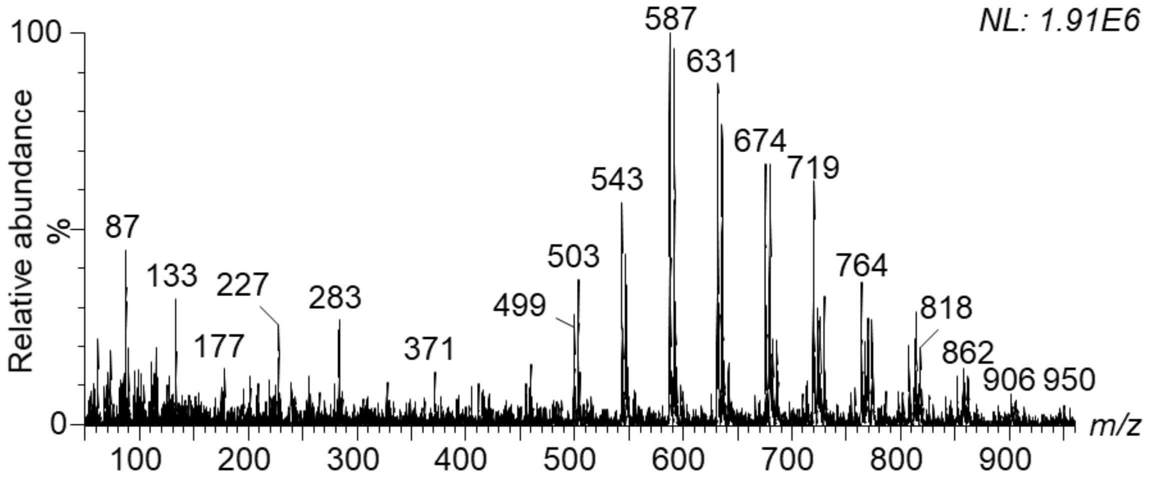

Figure S22 Nile Red stained polypropylene

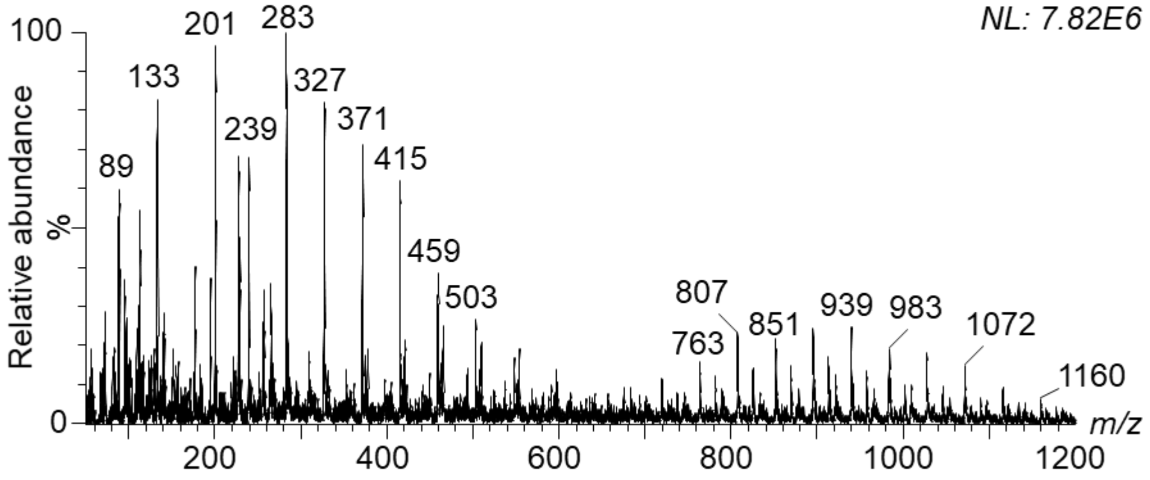

Figure S23 Tween20 0.2% in ultrapure water (1 µL)

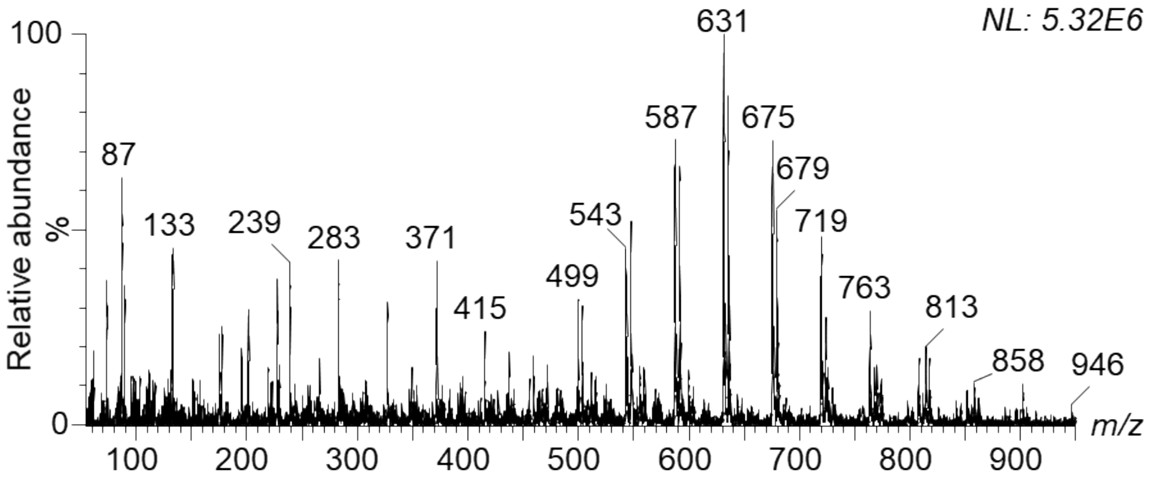

Supplement: Supplementary file 1 — ac2c04345_si_001.pdf [file ac2c04345_si_001.pdf]
